# Illumination levels in commonly used ophthalmic devices

**DOI:** 10.1007/s00417-023-06189-9

**Published:** 2023-08-07

**Authors:** Piotr Kanclerz, Natasza Bazylczyk

**Affiliations:** 1 Helsinki Retina Research Group, University of Helsinki, Helsinki, Finland; 2 Department of Ophthalmology, Hygeia Clinic, ul. Jaśkowa Dolina 57, 80-286 Gdańsk, Poland

**Keywords:** Pupil size, Pentacam, Optical coherence tomography, Autorefractometry, Scotopic pupil

## Abstract

Pupil diameter is a key parameter for corneal and multifocal intraocular lens surgery. Many devices are dedicated to measure the pupil size, but do not specify the illumination during capture. The aim of this study was to present illumination levels in routinely used ophthalmic devices which present pupil sizes. To obtain measurements, the lux meter was placed in the chin rest in the corneal plane and the room was completely dimmed. Ten measurements were taken for each device. The illumination levels for white and red Placido disk corneal topographers were 1253.1 ± 0.2 and 329.0 ± 0.2 lux, respectively (both photopic conditions). Scheimpflug corneal tomography should be considered as a mesopic measurement (14.5 ± 0.1 lux). Optical coherence tomography and autorefractometry are scotopic measurements (0.4–0.6 lux). We postulate that producers should provide illumination levels of their devices measuring pupil size. Moreover, when mentioning a pupil size, one should consider presenting to what lighting conditions it refers to.

**Supplementary Information:**

The online version contains supplementary material available at 10.1007/s00417-023-06189-9.


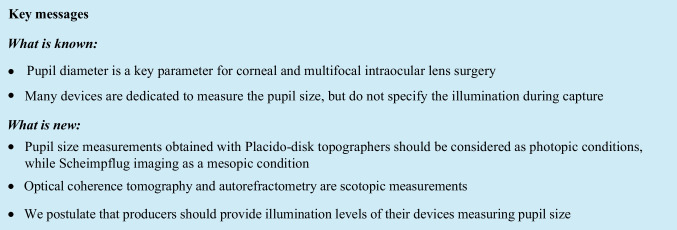


Dear Editor,

It is well known that pupil size depends on the illuminance level. Pupil diameter is a key parameter for corneal and multifocal intraocular lens surgery, as poor quality of twilight vision, halos and glare can be related to a large pupil size. Many devices (such as iTrace, OPD, OQAS) measure visual quality fluctuations at different pupil sizes, while several others are dedicated to measure the pupil size. However, the level of illumination during the capture is commonly not specified by the manufacturers. The aim of this study was to present illumination levels in routinely used ophthalmic devices which present pupil sizes.

For measurements, the Benetech GM1030 (Wintact Electronics Co., Shenzhen, China) lux meter was used. The lux meter was placed in the chin rest on a dedicated arm in the corneal plane (Supplementary Figure 1). The room was completely dimmed; the device was focused on the luxmeter as is done during clinical measurement. Ten measurements were made, and the mean maximum luminance during capture is presented in Table 1. Results show that measurements obtained with illuminated Placido-disk topographers should be considered as photopic conditions, while Scheimpflug imaging as a mesopic condition. Optical coherence tomography and autorefractometry could be considered as scotopic conditions.

**Table 1:** Illumination levels at the corneal level in commonly ophthalmic devices

Device	Measurement	Maximum illumination level
Oculus Pentacam AXL	Biometry and corneal tomography	14.5 ± 0.1 lux
Oculus Keratograph 3	Red placido-disk corneal topography	329.0 ± 0.2 lux
Oculus Keratograph 5M	White placido-disk corneal topography	1,253.1 ± 0.2 lux
Oculus Keratograph 5M	Low glare mode corneal topography	0.4 ± 0.0 lux
Nidek ARK-1a	Autorefractometry	0.6 ± 0.0 lux
Optopol Revo	Retinal optical coherence tomography	0.4 ± 0.0 lux

Illumination levels of different devices. A scotopic pupil is usually observed in 0.4 lux illumination, mesopic in 4 lux, and a photopic pupil in at least 40 lux illumination

Several devices are dedicated to measure the scotopic pupil size, i.e. a Colvard pupillometer, NeurOptics or Procyon devices [1]. However, this is the first study presenting illumination levels at the corneal plane for several commonly used ophthalmic devices. It is known that the pupil size decreases linearly with age [2]. A small pupil is observed in several medical conditions including diabetes, pseudoexfoliation syndrome, uveitis, or mature cataracts [2]. Patients with larger preoperative pupils can experience more photic phenomena and have lower satisfaction following cataract surgery. Interestingly, Koch et al. found that preoperative pupil size does not reliably predict postoperative pupil size after cataract surgery [3]. Still, multifocal intraocular lenses are not recommended in cases of large scotopic pupil diameter, especially for patients who often drive at night.

A large pupil diameter in patients undergoing corneal refractive surgery might be associated with visual disturbances, namely glare, haze and halos. Historically, the optical zone diameter should be at least as large as the pupil to preclude glare at the fovea [1]. In a newer review, Salz and Trattler suggested that the maximum scotopic pupil size should be not greater than 7 mm [1]. Schallhorn et al. found that although patients with mesopic large pupils commonly reported glares, haze and halos in the early postoperative period, the symptoms diminished 6 months after the procedure [4]. In another study, there was no correlation between large pupil size (≥6.5 mm) and postoperative visual symptoms 12 months after surgery [5]. Wavefront-guided ablations potentially reduce the prevalence of photic phenomena following corneal refractive surgery [1, 5]. Still, operating on patients with large scotopic pupils can be a contentious decision.

In conclusion, we postulate that producers should provide illumination levels of their devices measuring pupil size. Moreover, when mentioning a pupil size, one should consider presenting to what lighting conditions it refers to.

### Supplementary information

ESM 1 Supplementary Figure 1. Scheimpflug scans of the measurement device (PNG 1414 kb)
