# Supplementary figures and images for: Illumination levels in commonly used ophthalmic devices
Source: Graefes Arch Clin Exp Ophthalmol. 2023 Aug 7;262(3):995–6. doi: 10.1007/s00417-023-06189-9 (PMC10907405; doi:10.1007/s00417-023-06189-9)

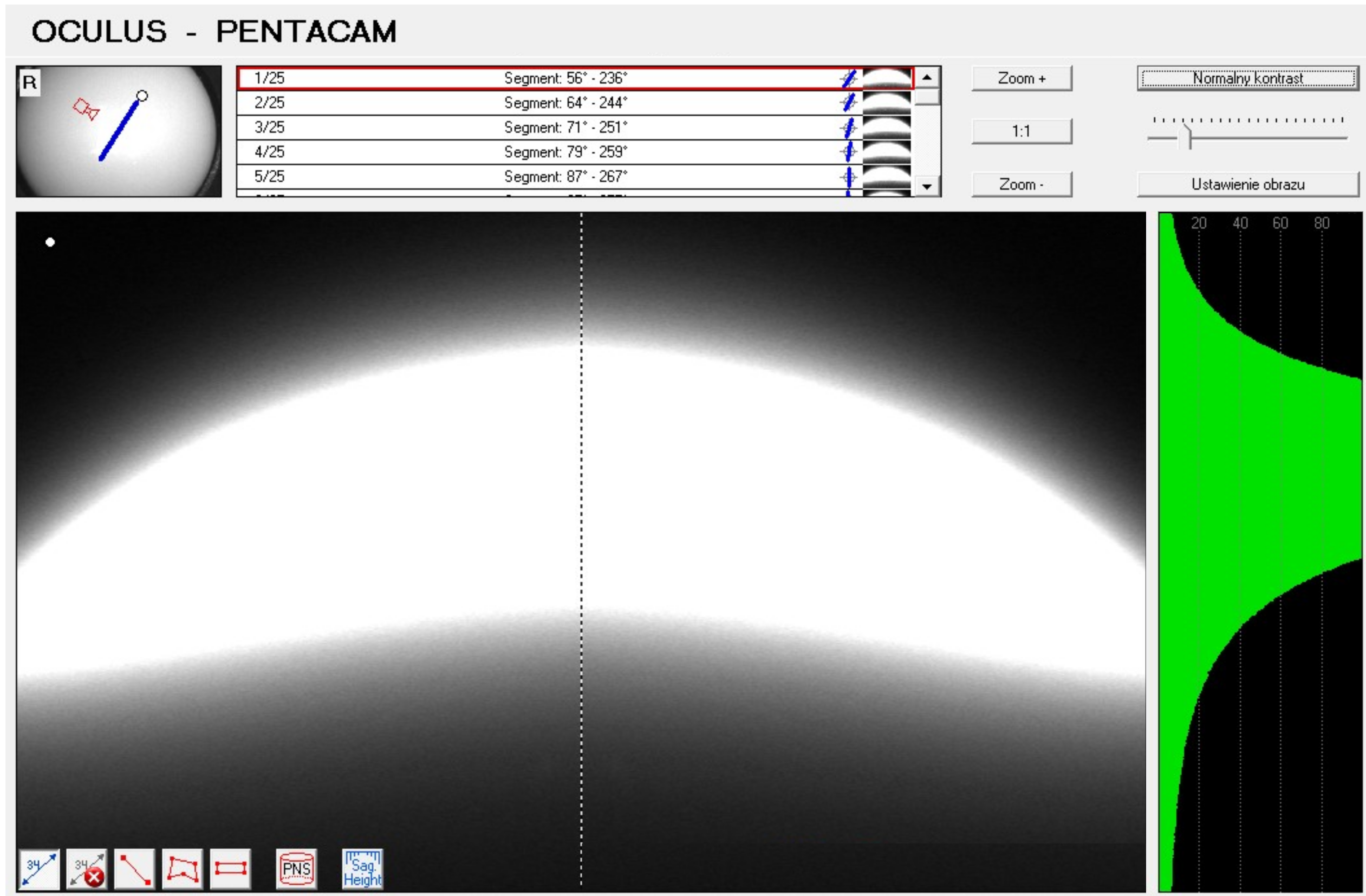

Supplement: Supplementary file 1 — Supplementary Figure 1. Scheimpflug scans of the measurement device (PNG 1414 kb) [file 417_2023_6189_MOESM1_ESM.png]
